# Synthesis of Poly(propylene oxide)–Poly(*N,N′*-dimethylacrylamide) Diblock Copolymer Nanoparticles via Reverse Sequence Polymerization-Induced Self-Assembly in Aqueous Solution

**DOI:** 10.1021/acs.macromol.3c01939

**Published:** 2023-12-17

**Authors:** Matthew A. H. Farmer, Osama M. Musa, Iris Haug, Stefan Naumann, Steven P. Armes

**Affiliations:** † Department of Chemistry, University of Sheffield, Dainton Building, Brook Hill, Sheffield, South Yorkshire S3 7HF, U.K.; ‡ Ashland Specialty Ingredients, 1005 US 202/206, Bridgewater, New Jersey 08807, United States; § Institute of Polymer Chemistry, University of Stuttgart, 70569 Stuttgart, Germany

## Abstract

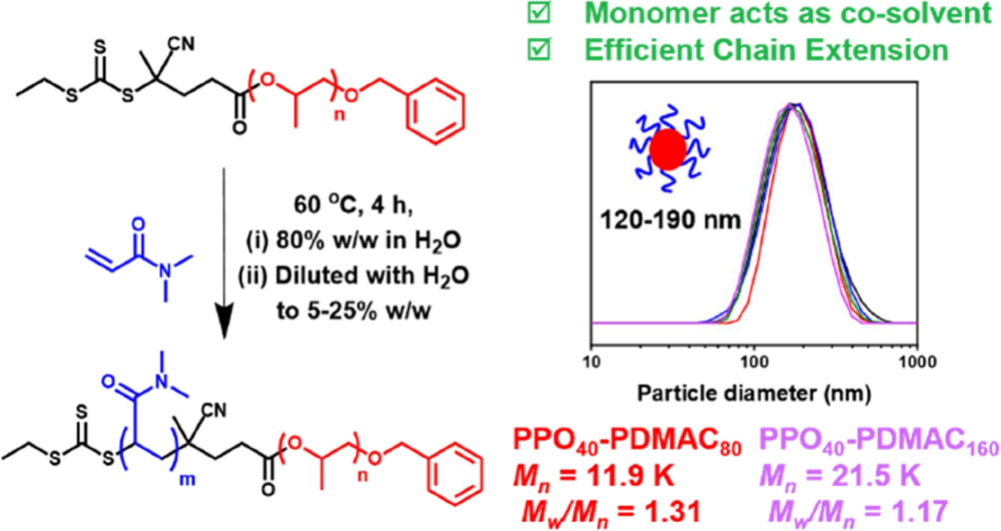

Sterically-stabilized diblock copolymer nanoparticles comprising poly(propylene oxide) (PPO) cores are prepared via reverse sequence polymerization-induced self-assembly (PISA) in aqueous solution. *N,N*′-Dimethylacrylamide (DMAC) acts as a cosolvent for the weakly hydrophobic trithiocarbonate-capped PPO precursor. Reversible addition–fragmentation chain transfer (RAFT) polymerization of DMAC is initially conducted at 80% w/w solids with deoxygenated water. At 30–60% DMAC conversion, the reaction mixture is diluted to 5–25% w/w solids. The PPO chains become less solvated as the DMAC monomer is consumed, which drives in situ self-assembly to form aqueous dispersions of PPO-core nanoparticles of 120–190 nm diameter at 20 °C. Such RAFT polymerizations are well-controlled (*M*_w_/*M*_n_ ≤ 1.31), and more than 99% DMAC conversion is achieved. The resulting nanoparticles exhibit thermoresponsive character: dynamic light scattering and transmission electron microscopy studies indicate the formation of more compact spherical nanoparticles of approximately 33 nm diameter on heating to 70 °C. Furthermore, 15–25% w/w aqueous dispersions of such nanoparticles formed micellar gels that undergo thermoreversible (de)gelation on cooling to 5 °C.

## Introduction

Over the past two decades, polymerization-induced self-assembly (PISA) has become widely recognized as an efficient synthetic route to a wide range of block copolymer nano-objects.^1−14^ Such nanoparticles offer various potential applications, including 3D cell culture media, thermoresponsive hydrogels for the long-term storage of human stem cells, nanoparticle lubricants, viscosity modifiers, opacifiers for paints, and bespoke Pickering emulsifiers.^15−20^

One important advantage of PISA is its versatility: such syntheses can be conducted in either polar or non-polar solvents.^21−30^ In the case of aqueous formulations, PISA typically involves growing a water-insoluble block from one end of a water-soluble precursor block.^23,31−34^ The resulting amphiphilic diblock copolymer chains undergo self-assembly at some critical degree of polymerization (DP) to form sterically-stabilized nano-objects.^35−37^ At first sight, it appears to be axiomatic that the water-soluble precursor should be prepared first because this block confers colloidal stability via steric stabilization.^38^ However, we have recently challenged this restrictive paradigm by developing new reverse sequence aqueous PISA formulations.^39−41^ In each case, the key step is the initial synthesis of relatively large charge-stabilized latex particles via reversible addition–fragmentation chain transfer (RAFT)^42−46^ aqueous dispersion polymerization of either 2-hydroxypropyl methacrylate (HPMA)^39,40^ or 4-hydroxybutyl acrylate (HBA).^41^ This involves the judicious choice of a suitable ionic RAFT agent to confer either anionic or cationic surface groups. In the second step, PHPMA latex particles act as a locus for the RAFT polymerization of either a suitable hydrophilic (meth)acrylic monomer or a protected hydrophobic monomer that can be subsequently rendered hydrophilic via in situ acid hydrolysis.^39,40^ Alternatively, the PHBA latex can be dissolved using a suitable water-miscible monomer such as *N*-(2-acryloyloxyethyl) pyrrolidone (NAEP) such that the subsequent RAFT polymerization is initially conducted in aqueous solution.^41^ Ultimately, both routes lead to the formation of much smaller sterically-stabilized diblock copolymer nanoparticles. Furthermore, we recently reported the efficient synthesis of PCL-core nanoparticles by reverse sequence PISA synthesis.^47^ In this case, various PCL precursors were dissolved as a concentrated aqueous solution using an alternative hydrophilic monomer, *N,N*′-dimethylacrylamide (DMAC), as a cosolvent. Subsequently, RAFT polymerization followed by in situ dilution with water led to the formation of well-defined sterically-stabilized spherical nanoparticles that underwent hydrolytic degradation under relatively mild conditions.^47^

**Scheme 1:**
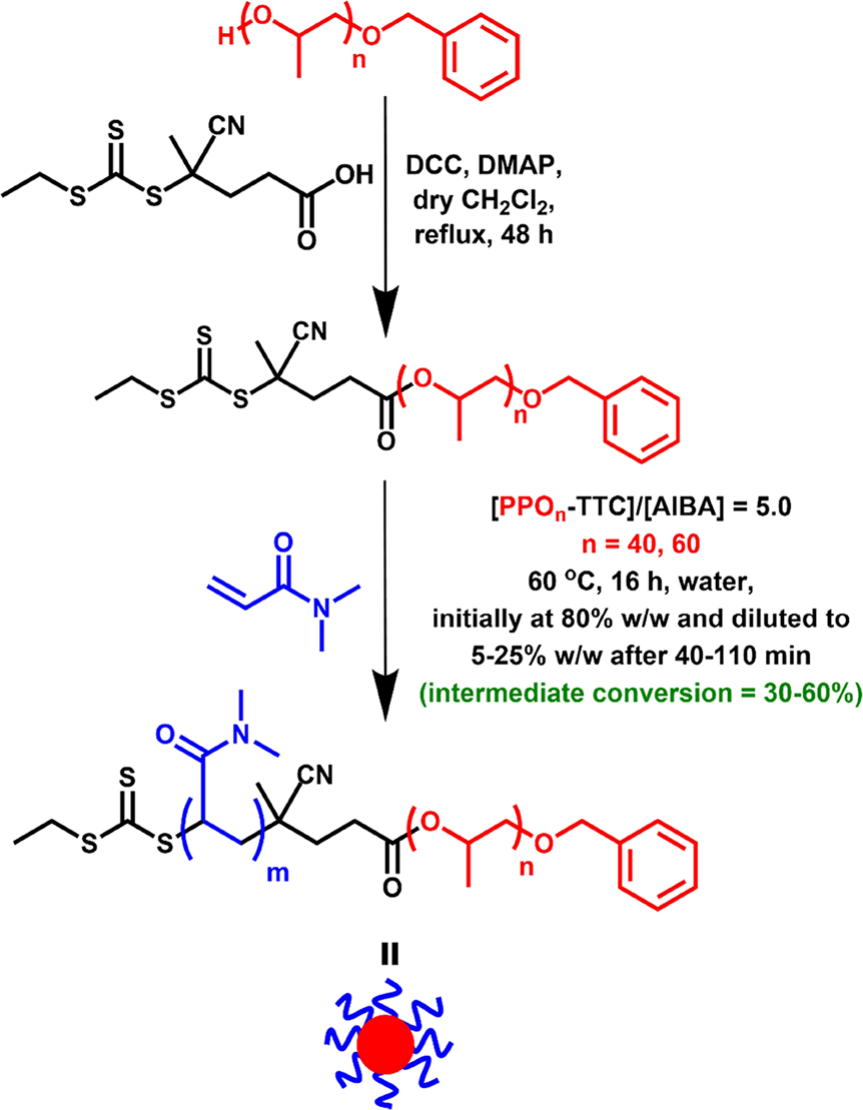
Esterification of a Monohydroxy-Capped PPO Precursor Using a Carboxylic Acid-Functionalized RAFT Agent (CEPA) via DCC/DMAP Chemistry Subsequently, RAFT polymerization of DMAC is conducted in a concentrated aqueous solution (80% w/w solids) at 60 °C. At a suitable intermediate DMAC conversion, dilution to 5–25% w/w solids using deionized water leads to in situ self-assembly of the amphiphilic PPO–PDMAC chains to produce sterically stabilized diblock copolymer nanoparticles with PPO cores.

Herein we report a new reverse sequence aqueous PISA formulation. In this case, we use the water-miscible monomer DMAC as a cosolvent for a weakly hydrophobic trithiocarbonate-capped poly(propylene oxide) (PPO-TTC) precursor in concentrated aqueous solution. RAFT polymerization of DMAC leads to the formation of amphiphilic PPO–PDMAC diblock copolymer chains and, at some critical monomer conversion, there is no longer sufficient DMAC cosolvent to ensure solubilization of the PPO chains. This results in in situ self-assembly to form nascent PPO-core nanoparticles, and the steric stabilizer chains subsequently grow longer as the remaining DMAC monomer is polymerized (see Scheme 1). This scenario differs from that of conventional PISA syntheses in that polymerization does not take place within monomer-swollen particles.^37^ Importantly, it is well documented that the PPO block undergoes degradation under appropriate conditions.^48−51^ Thus, this reverse sequence aqueous PISA formulation offers a route to new degradable diblock copolymer nanoparticles. This approach offers several advantages compared to the (co)polymerization of various cyclic monomers as recently developed by others.^52−63^ For example, cyclic monomers suitable for radical ring-opening polymerization require inefficient multistep syntheses while the synthesis of *N*-carboxyanhydrides requires the use of hazardous chemicals (e.g., phosgene).^64^

## Experimental Section

### Materials

Unless otherwise stated, all reagents were used as received. 2,2′-Azobis(2-methylpropionamidine) dihydrochloride (AIBA; 97%), *N,N*′-dicyclohexylcarbodiimide (DCC; 99%), *N,N*′-dimethylacrylamide (DMAC; 99%), anhydrous magnesium sulfate, D_2_O (99.9%), lithium bromide, and *n*-hexane were purchased from Sigma-Aldrich (Dorset, U.K.). 4-(Dimethylamino)pyridine (DMAP) was purchased from Alfa Aesar (Heysham, U.K.). Dimethylformamide (DMF) was purchased from VWR (Leicestershire, U.K.). Ethyl acetate was purchased from Fisher Scientific (Loughborough, U.K.). Deuterated methanol (CD_3_OD, 99.8%) and deuterated dichloromethane (99.8%) were purchased from Goss Scientific Instruments Ltd. (Cheshire, U.K.). Hydroquinone was purchased from Scientific Laboratory Supplies Ltd. (Nottingham, U.K.). The two monohydroxy-capped poly(propylene oxide) (PPO) precursors used in this work were prepared via anionic polymerization of propylene oxide using an organocatalyst to ensure near-monodisperse PPO chains and high end-group fidelity.^65,66^ Their mean DPs were determined to be 40 and 60, respectively, by end-group analysis using ^1^H NMR spectroscopy (Figures S1 and S2). The RAFT agent 4-cyano-4-(ethylsulfanylthiocarbonyl)sulfanyl pentanoic acid (CEPA) was prepared in 82% yield according to a literature protocol.^67^ For synthesis of the trithiocarbonate-capped PPO precursors, anhydrous dichloromethane was obtained from an in-house Grubbs purification solvent system. Deionized water was obtained from an Elgastat Option 3A water purification unit (resistivity = 15 MΩ cm).

### Characterization Techniques

#### ^1^H Nuclear Magnetic Resonance Spectroscopy

A 400 MHz Bruker Avance-400 spectrometer was used to obtain ^1^H NMR spectra at 298 K, with 16 scans averaged per spectrum. For kinetic experiments, aliquots of the reaction mixture were diluted using CD_3_OD, and DMAC conversions were calculated by comparing the integrated vinyl proton signals at 6.75, 6.20, and 5.76 ppm to the aromatic signal at 7.37 ppm assigned to the PPO_40_-TTC precursor. NMR spectra for the RAFT agent, the two PPO precursors, and final diblock copolymers were recorded in CD_2_Cl_2_ to ensure that the solvent (and residual water) signals did not overlap with the polymer signals. Aqueous dispersions of diblock copolymer nanoparticles were dissolved in CD_2_Cl_2_ prior to drying with anhydrous magnesium sulfate. Each solution was then passed through a 0.20 μm filter prior to NMR analysis. Variable temperature ^1^H NMR studies were performed on an aqueous dispersion of PPO_60_–PDMAC_120_ nanoparticles after dilution from 10% w/w to 1% w/w using D_2_O. In this case, ^1^H NMR spectra were recorded using a 500 MHz Bruker Avance III HD spectrometer, with 16 scans being averaged per spectrum. Samples were allowed to equilibrate for 10 min at each temperature prior to spectral acquisition.

#### Gel Permeation Chromatography

Number-average molecular weights (*M*_n_), weight-average molecular weights (*M*_w_), and dispersities (*M*_w_/*M*_n_) were determined using an Agilent 1260 Infinity GPC system operating at 60 °C and equipped with two Agilent PL-gel 5 μm Mixed-C columns, a guard column, a differential refractive index detector, and a UV–visible detector operating at 305 nm. Unless otherwise stated, the eluent was HPLC-grade DMF containing 10 mM LiBr and a flow rate of 1.0 mL min^–1^. For GPC analysis of the PPO-TTC precursors, LiBr was omitted from the eluent because the presence of this salt produced a signal that overlaps with the polymer signal (see Figure S3). A series of near-monodisperse poly(methyl methacrylate) polymers with *M*_p_ values ranging from 800 to 2,200,000 g mol^–1^ was used for calibration. Samples were prepared for GPC analysis by diluting using the eluent, and chromatograms were analyzed using Agilent GPC/SEC software.

#### Dynamic Light Scattering

DLS analysis was performed on 1.0% w/w aqueous copolymer dispersions prepared by diluting the original dispersion to 1.0% w/w using deionized water. Unless stated otherwise, a Malvern Instruments Zetasizer Nano ZS instrument equipped with a 4 mW He–Ne laser (λ = 633 nm) was used to obtain DLS data at 20 °C to obtain the DLS data. Scattered light was detected using an avalanche photodiode detector at 173°. Each sample was equilibrated for 5 min prior to measurements. The z-average particle diameter (*D*_z_), number-average particle diameter (*D*_n_), and polydispersity index (PDI) were averaged over three consecutive runs consisting of ten measurements per run.

When targeting a PPO_60_–PDMAC_120_ diblock copolymer, the polymerization was quenched at 50% DMAC conversion (as judged by ^1^H NMR spectroscopy) by exposing the reaction mixture to air while cooling with the aid of an ice bath. Subsequently, the reaction mixture was diluted to 1.0% w/w solids using either 60% w/w aqueous DMAC (to examine whether nanoparticles were present immediately prior to dilution) or 4% w/w aqueous DMAC (to examine whether the nanoparticles formed immediately after dilution owing to the ensuing change in solvency). A free radical inhibitor (hydroquinone, 18.0 mM) was added to each dilute aqueous solution, which was also oxygenated via air sparging for 15 min to prevent any further polymerization. In each case, DLS analysis was performed at 60 °C after allowing 10 min for thermal equilibration.

#### Transmission Electron Microscopy (TEM)

A 10 μL droplet of a 1.0% w/w aqueous copolymer dispersion was placed onto a copper/palladium grid (Agar Scientific, U.K.) that had been previously coated in-house with a thin film of amorphous carbon and then subjected to a plasma glow-discharge for 30 s to generate a hydrophilic surface. After 1 min, the aqueous droplet was blotted to remove excess solution copolymer. A 0.75% w/v aqueous solution of uranyl formate was employed as a negative stain to enhance electron contrast. Accordingly, a 10 μL droplet of aqueous uranyl formate solution was placed on the grid for 20 s and then blotted to remove excess. The grid was then carefully dried using a vacuum hose. When preparing TEM grids for aqueous nanoparticle dispersions dried at 70 °C, each 1% w/w aqueous dispersion was heated in a 70 °C oven for 30 min prior to rapid grid preparation (<1 min). Imaging was performed using a FEI Tecnai Spirit 2 microscope equipped with an Orius SC1000B camera operating at 80 kV.

#### Rheology

An Anton Paar MCR 502 rheometer equipped with a variable temperature Peltier plate/hood and a 50 mm 2° stainless steel cone was used for all experiments. Loss and storage moduli were determined as a function of temperature at a fixed strain of 0.5% and a shear rate of 1.0 rad s^–1^ at a heating/cooling rate of 2 °C min^–1^.

#### Shear-Induced Polarized Light Imaging (SIPLI)

Shear alignment experiments were conducted using an Anton Paar Physica MCR301 rheometer equipped with a SIPLI attachment. For these measurements, 25 mm polished steel and fused quartz plates were employed using a plate–plate geometry with a zero gap of 0.50 mm. Temperature was controlled using a Peltier system with a Peltier hood. Sample illumination was achieved using a high-intensity fiber optic white light source (150 W MI-150) supplied by Edmund Optics (York, U.K.). Polarized light images were recorded with the polarizer and analyzer axes crossed at 90° using a color CCD camera (Lumenera Lu165c).

#### Aqueous Electrophoresis

Measurements were performed on 1.0% w/w aqueous copolymer dispersions using a Malvern Instruments Zetasizer Nano ZS instrument. Concentrated copolymer dispersions were diluted to 1.0% w/w using an aqueous solution of 1 mM KCl, which served as a background electrolyte. Either 0.1 M NaOH or 0.1 M HCl was used to adjust the solution pH. In all cases, electrophoretic mobilities were determined at 20 °C, and the Henry equation was used to calculate zeta potentials using the Smoluchowski approximation.

### Synthetic Protocols

#### Synthesis of the PPO-TTC Precursor

DMAP (47.8 mg, 0.39 mmol), DCC (1.44 g, 6.97 mmol), monohydroxy-capped PPO_40_ (5.00 g, 2.06 mmol), and CEPA (0.91 g, 3.49 mmol) were weighed into four 28 mL glass vials in turn that had been previously dried in a 200 °C oven overnight. Each vial was sealed using a rubber septum and then placed in a vacuum oven for 2 h at 35 °C to dry each reagent. Subsequently, a minimal amount of dry CH_2_Cl_2_ was used to dissolve each reagent in each vial. A dry two-necked 100 mL round-bottom flask containing a magnetic stirrer bar was fitted with a condenser, sealed with two rubber septa and then charged with CH_2_Cl_2_ solutions containing DMAP, CEPA, and monohydroxy-capped PPO_40_. The resulting solution was stirred over ice, and the CH_2_Cl_2_ solution containing DCC was added dropwise to the round-bottom flask. This reaction mixture was heated to reflux while purging with nitrogen gas and then stirred for 48 h at reflux.

Crude PPO_40_-TTC was purified by column chromatography (with silica gel as the stationary phase) using a 60:40 ethyl acetate/*n*-hexane mobile phase to remove the first fraction. Pure ethyl acetate was used to remove the second fraction, which was dried under reduced pressure to produce a viscous orange oil. The purified precursor contained no residual CEPA starting material as judged by UV GPC analysis. End-group analysis by ^1^H NMR spectroscopy, comparing involved the integrated proton signal at 1.91 ppm assigned to the methyl group of the RAFT agent with the unique PPO backbone signal at 3.55 ppm and pendant methyl group at 1.16 ppm. This approach indicated a degree of esterification of 97 ± 1% for PPO_40_-TTC (see Figure S4). The same approach was used to prepare PPO_60_-TTC, with end-group analysis indicating a degree of esterification of 97 ± 1% in this case (see Figure S5).

#### RAFT Polymerization of DMAC in Concentrated Aqueous Solution Using PPO_40_-TTC with Subsequent Dilution at an Intermediate DMAC Conversion

A 14 mL glass vial containing a magnetic flea was charged with PPO_40_-TTC (0.10 g, 0.038 mmol), DMAC (0.45 g, 4.52 mmol, target DP = 120), and an aqueous AIBA solution (0.14 mL of a 54 mM solution, or 7.5 μmol AIBA; PPO_40_-TTC/AIBA molar ratio = 5) to target 80% w/w solids. After sealing with a rubber septum, this vial was placed in an ice bath, and the reaction mixture was deoxygenated with N_2_ gas for 30 min. The vial was then immersed in an oil bath set at 60 °C. The stirred reaction mixture became much more viscous after 40 min owing to the DMAC polymerization. At this point, deoxygenated water (4.8 mL, preheated to 60 °C, targeting 10% w/w solids) was added using a degassed needle and syringe. An aliquot of the reaction mixture was extracted, and ^1^H NMR spectroscopy analysis indicated that a DMAC conversion of 38% had been achieved. The polymerization was allowed to proceed for 16 h at 60 °C and then quenched by exposing the reaction mixture to air while cooling to 20 °C. A final DMAC conversion of more than 99% was determined by ^1^H NMR analysis. For analogous syntheses using alternative PPO DPs and targeting different PDMAC DPs, the reagent quantities and volume of water were adjusted accordingly (see Table S1 for further details).

## Results and Discussion

Recently, we reported the synthesis of hydrolytically degradable poly(ε-caprolactone)–poly(*N,N*′*-*dimethylacrylamide) nanoparticles via reverse sequence PISA in aqueous media.^47^ This represents an interesting alternative approach to the PISA synthesis of hydrolytically degradable nanoparticles via statistical copolymerization of a vinyl monomer with various cyclic monomers developed by other research groups.^53,55,60^ Herein, we expand on this new strategy using poly(propylene oxide) (PPO) as a weakly hydrophobic precursor for reverse sequence PISA, as detailed in Scheme 1. Notably, PPO is a cost-effective polymer that is manufactured on an industrial scale: it is widely used as a lubricant or rheology modifier and acts as an important component for polyurethane foams and *Pluronics*.^68,69^ Importantly, PPO undergoes (bio)degradation under universally recognized test conditions developed by the Organization for Economic Cooperation and Development (OECD).^48−51^ More specifically, hydroxy-capped PPO homopolymer with a mean DP of 34 is known to be biodegradable: a theoretical oxygen demand of 105 ± 1% is observed within 28 days according to an OCED-approved biodegradability test, which suggests. This suggests a half-life of 1.6 ± 0.4 days.^48^ Furthermore, PPO with a mean DP of 46 also undergoes biodegradation under the same conditions, albeit at a slower rate: a theoretical oxygen demand of 32 ± 7% was observed within 28 days, with a corresponding half-life of 24 ± 5 days.^48^ A well-known prerequisite for such degradation is the presence of a terminal hydroxyl group.^70^ In the present study, the PPO precursor is linked to its trithiocarbonate end-group by an ester bond. Thus, initial hydrolysis of this ester produces a hydroxy-capped PPO, which should then undergo subsequent biodegradation.

**Figure 1:**
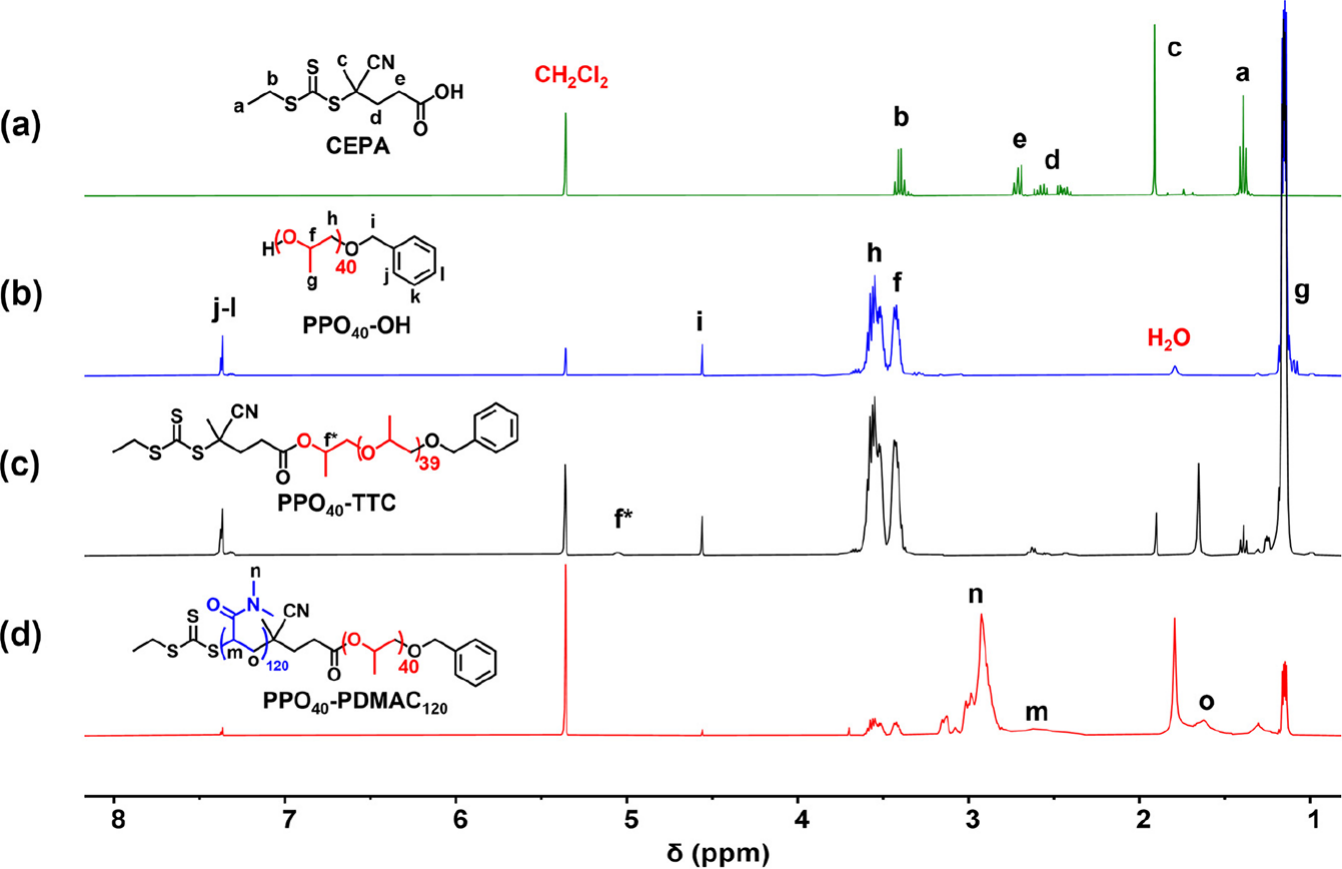
^1^H NMR spectra (CD_2_Cl_2_) recorded for (a) CEPA RAFT agent, (b) monohydroxy-capped PPO_40_ precursor, (c) monofunctional PPO_40_-TTC RAFT agent (where f* represents the oxymethine ester proton), and (d) PPO_40_-PDMAC_120_ diblock copolymer.

Two monofunctional trithiocarbonate-capped PPO RAFT agents were prepared by esterification of a monohydroxy-capped PPO precursor using a carboxylic acid-functionalized RAFT agent (CEPA).^71^ The mean degree of esterification was determined by ^1^H NMR spectroscopy by integrating the PPO proton signals at 3.55 and 1.16 ppm (corresponding to the unique PPO backbone signal and the pendent methyl group) with respect to the methyl proton signal c at 1.91 ppm (see Figure 1). The mean degree of esterification for the PPO_40_-TTC and PPO_60_-TTC precursors was determined to be 97 ± 1 and 97 ± 1%, respectively (see Figures S4 and S5). Furthermore, UV GPC analysis (λ = 305 nm) confirmed that no residual CEPA starting material remained after purification of each PPO-TTC precursor via column chromatography (see Figure S6).

The water-miscible DMAC monomer acts as a cosolvent for the PPO-TTC, enabling initial solubilization of this weakly hydrophobic precursor in hot concentrated aqueous solution (e.g., 80% w/w solids). Subsequently, the RAFT polymerization of DMAC was conducted at 60 °C. As the DMAC monomer is gradually consumed, the reaction mixture inevitably became a poorer solvent for the PPO chains. At some intermediate DMAC conversion, a significant increase in solution viscosity is observed. At this point, the reaction mixture is diluted with deoxygenated deionized water to the desired final copolymer concentration. At full DMAC conversion, this produces an aqueous dispersion of sterically-stabilized PPO–PDMAC nanoparticles comprising PPO cores and PDMAC coronas (see Scheme 1).

**Figure 2:**
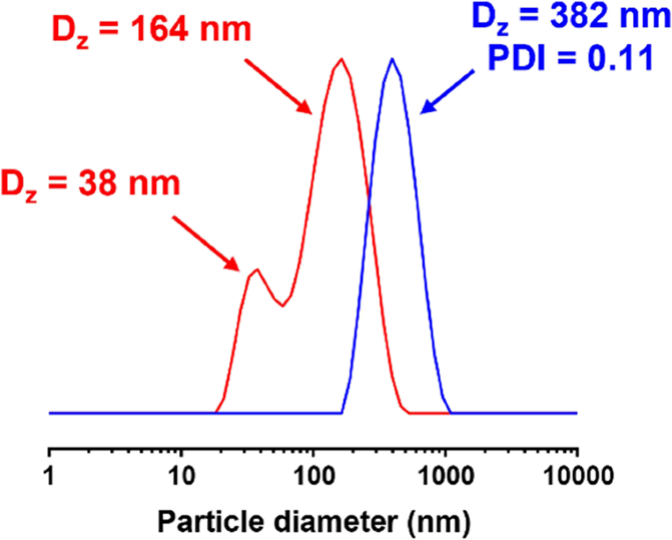
DLS analysis of a reverse sequence aqueous PISA formulation targeting PPO_60_–PDMAC_120_ after quenching at 81 min, which is the time point chosen for the dilution of this reaction mixture with deionized water. DLS analysis is performed at 60 °C after dilution to 1% w/w solids using either 60% w/w aqueous DMAC monomer (blue curve) or 4% w/w aqueous DMAC monomer (red curve). See main text for further details.

Visual inspection of the reaction mixture confirms an increase in turbidity immediately prior to dilution, which suggests the onset of aggregation. In principle, time-resolved small-angle X-ray scattering (SAXS) studies of the concentrated reaction mixture should enable the onset of self-assembly to be monitored. Unfortunately, such experiments would require access to a synchrotron facility and are beyond the scope of the current study. Instead, we examined whether aggregation occurs prior to dilution by quenching a reaction mixture targeting PPO_60_–PDMAC_120_ nanoparticles after 81 min, which corresponds to the time point at which this formulation was diluted with deionized water. ^1^H NMR spectroscopy studies indicated a DMAC conversion of 50% at 81 min. Hence the reaction mixture comprised 50% w/w PPO_60_–PDMAC_60_, 30% w/w DMAC monomer, and 20% w/w water. This quenched reaction mixture was then diluted to 1.0% w/w using a 60% w/w aqueous DMAC solution for DLS analysis. This protocol was used to ensure that dilution did not lead to a change in the solvent quality, which would most likely disrupt the nascent aggregates formed prior to quenching the polymerization. DLS analysis performed at the reaction temperature of 60 °C indicated a z-average diameter of 388 nm, which suggests the formation of relatively ill-defined loose aggregates prior to dilution (see Figure 2). Immediately after dilution, the reaction mixture comprises 6% w/w PPO_60_–PDMAC_60_, ∼4% w/w DMAC monomer, and 90% w/w water. Thus, 4% w/w aqueous DMAC was used to further dilute this reaction mixture to 1.0% w/w copolymer for DLS analysis at 60 °C. A bimodal particle size distribution was observed, comprising a major population with a z-average diameter of 164 nm and a minor population with a z-average diameter of 38 nm (Figure 2). Moreover, the scattered light intensity increased more than six-fold compared to that observed prior to dilution, which suggests that more compact aggregates are formed after dilution. This is physically reasonable because dilution leads to a reduction in solvency for the weakly hydrophobic PPO chains. Finally, when this reverse sequence aqueous PISA formulation was allowed to proceed to full DMAC conversion, a bimodal particle size distribution was observed at 60 °C (see Figure S7).

**Figure 3:**
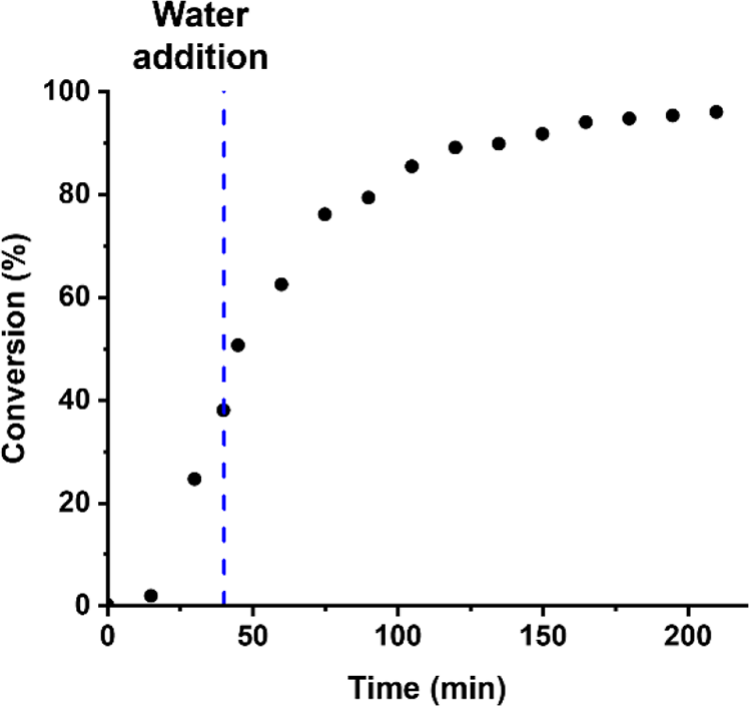
Conversion vs time curve obtained by ^1^H NMR spectroscopy for the reverse sequence aqueous PISA synthesis of PPO_40_–PDMAC_120_ nanoparticles at 60 °C. Initially, the RAFT polymerization of DMAC was conducted at 80% w/w solids with subsequent dilution to 10% w/w solids using deoxygenated deionized water after 40 min (which corresponds to 38% DMAC conversion). A final DMAC conversion of 96% was achieved within 210 min at 60 °C. Conditions: [PPO_40_-TTC]/[AIBA] molar ratio = 5.0.

Kinetic analysis was performed when using a PPO_40_-TTC precursor to target a PDMAC DP of 120 at 60 °C. After 40 min, the concentrated aqueous reaction mixture was diluted from 80% w/w to 10% w/w using deionized water. At this point, an intermediate conversion of 38% was achieved, as judged by ^1^H NMR spectroscopy. Subsequently, 96% DMAC conversion was achieved within a total reaction time of 210 min (Figure 3).

**Figure 4:**
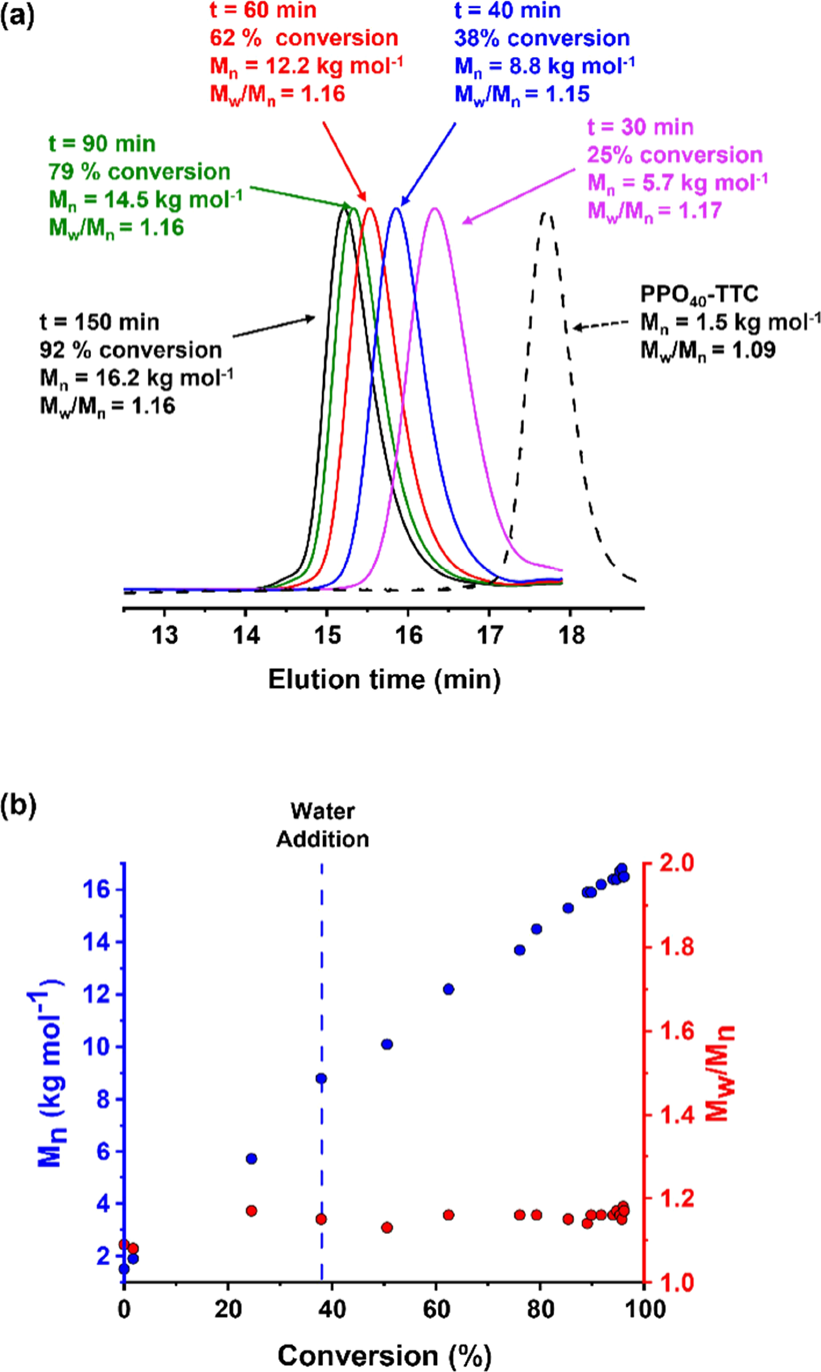
(a) Selected DMF GPC curves (refractive index detector) and (b) corresponding *M*_n_ (blue points) and *M*_w_/*M*_n_ (red points) data determined during the reverse sequence aqueous PISA synthesis of PPO_40_–PDMAC_120_ nanoparticles prepared at 60 °C. Initially, the RAFT polymerization of DMAC was conducted at 80% w/w solids, with subsequent dilution to 10% w/w solids using deoxygenated deionized water after 40 min (which corresponds to ∼38% DMAC conversion). Conditions: [PPO_40_-TTC]/[AIBA] molar ratio = 5.0 [N.B. diblock copolymer GPC curves are only shown up to an elution time of 17.9 min to omit the signal attributed to LiBr (see Figure S3). GPC analysis of the PPO precursor was performed in the absence of any LiBr salt].

GPC analysis indicated a linear evolution in molecular weight (*M*_n_) with conversion and narrow molecular weight distributions (*M*_w_/*M*_n_ < 1.20) throughout the DMAC polymerization, which suggests good RAFT control (see Figure 4). In addition, a relatively high blocking efficiency is obtained: there is little or no evidence for contamination of the PPO–PDMAC diblock copolymer chains by the PPO precursor. For traditional PISA formulations, a significant increase in the rate of polymerization typically occurs after micellar nucleation owing to the high local monomer concentration within the growing nanoparticles.^37^ However, this is not observed for the reverse sequence aqueous PISA formulations described herein simply because the RAFT chain-ends are located at the end of the PDMAC steric stabilizer chains, rather than within nanoparticle cores. However, it is perhaps worth emphasizing that the dilution of the polymerizing reaction mixture does not lead to a slower rate of polymerization. In fact, the semilogarithmic plot for the same kinetic data set indicates that the rate of polymerization before and after dilution is essentially identical because there is no discernible change in gradient (see Figure S8). This is presumably because acrylamide-based monomers polymerize significantly faster in dilute aqueous solution than in the bulk, as reported by Büback and co-workers.^72,73^

To further optimize this reverse sequence aqueous PISA formulation, the solids content was systematically varied from 60 to 90% w/w when targeting PPO_60_–PDMAC_120_. For syntheses targeting either 60 or 70% w/w solids, the reaction mixture became much more viscous after 35 min (68% DMAC conversion) or 60 min (52% DMAC conversion), respectively. However, dilution with deionized water led to immediate precipitation in both cases. Furthermore, visual inspection confirmed that a homogeneous aqueous solution was never achieved prior to dilution. In contrast, a homogeneous reaction mixture was formed within 5 min at 60 °C when targeting 80% w/w solids. Moreover, a stable colloidal dispersion was obtained after dilution of this reaction mixture with deionized water after 80 min (which corresponded to 49% DMAC conversion). On the other hand, the DMAC polymerization proceeded much more slowly when targeting 90% w/w solids: a discernible increase in the viscosity of the reaction mixture was only observed after 220 min at 60 °C (which corresponds to 32% DMAC conversion). This slower rate of polymerization is attributed to the reduced solubility of the AIBA initiator at 90% w/w solids (owing to the lower water content). Moreover, GPC analysis indicated final copolymer dispersities of 1.20 and 1.29 when targeting 80% and 90% w/w solids, respectively. Furthermore, UV GPC analysis (λ = 305 nm) indicated a significantly lower chain extension efficiency for the 90% w/w synthesis (see Figure S9). In summary, these additional experiments indicate that targeting 80% w/w solids is optimal because it enables a fast rate of polymerization to be achieved while also producing a stable colloidal dispersion.

**Figure 5:**
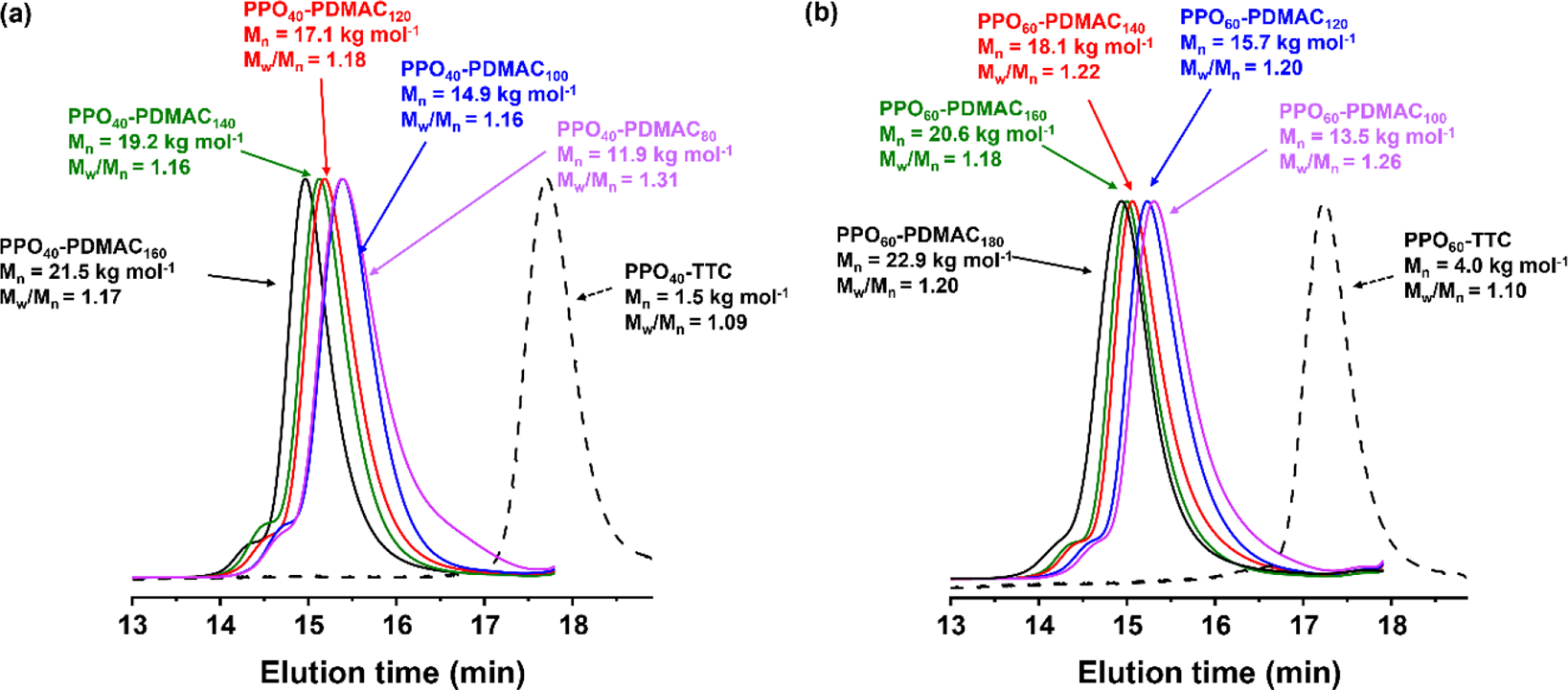
DMF GPC curves recorded using a refractive index detector for a series of PPO–PDMAC diblock copolymers prepared by reverse sequence aqueous PISA at 60 °C. (a) PPO_40_-TTC precursor and a corresponding series of PPO_40_–PDMAC_*n*_ (*n* = 80–160) diblock copolymers. (b) PPO_60_-TTC precursor and a corresponding series of PPO_60_–PDMAC_*m*_ (*m* = 100–180) diblock copolymers. Conditions: [PPO_*n*_-TTC]/[AIBA] molar ratio = 5.0 [N.B. diblock copolymer GPC curves are only shown up to an elution time of 17.9 min to omit the signal attributed to LiBr (see Figure S3). GPC analysis of the PPO precursor was performed in the absence of any LiBr salt].

Having established that such reverse sequence aqueous PISA formulations were well-controlled, a range of PDMAC DPs were targeted using the PPO_40_-TTC and PPO_60_-TTC precursors (see Figure 5). In all cases, ^1^H NMR spectroscopy studies indicated that essentially full DMAC conversion (>99%) was achieved within 16 h at 60 °C. Moreover, narrow molecular weight distributions and a linear increase in molecular weight with increasing target PDMAC DP were observed.

**Figure 6:**
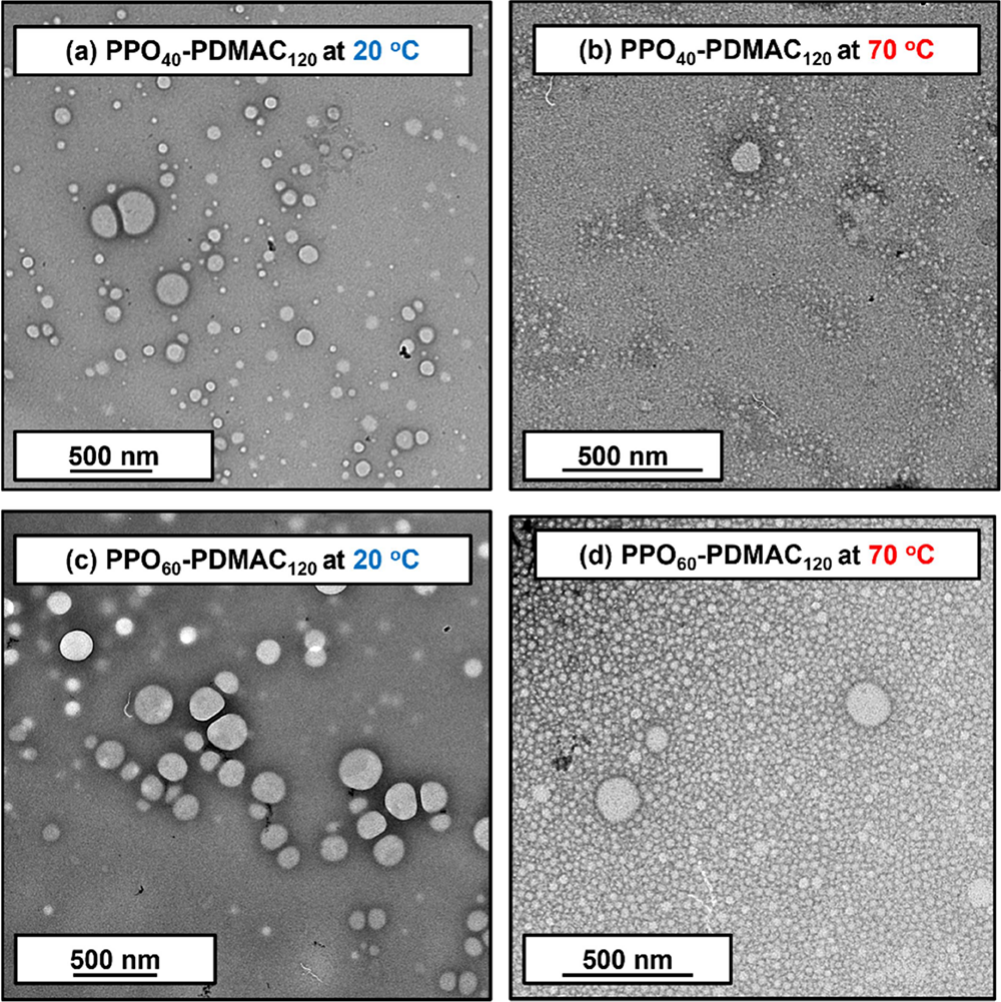
(a–d) Representative TEM images recorded after drying dilute aqueous dispersions of PPO_40_–PDMAC_120_ and PPO_60_–PDMAC_120_ nanoparticles at either 20 or 70 °C, respectively.

**Figure 7:**
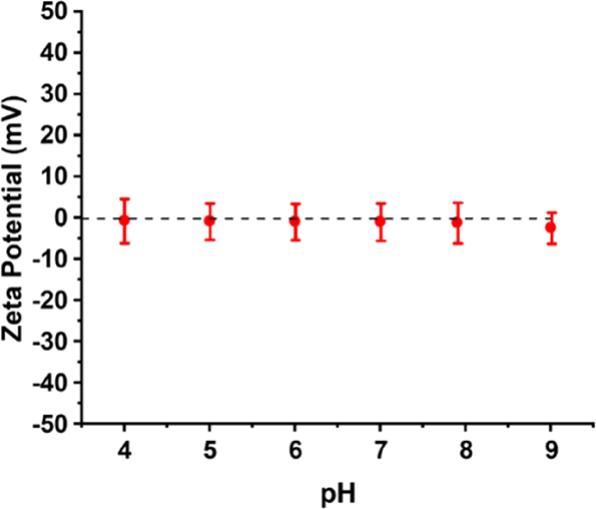
Zeta potential vs pH data obtained for a 1.0% w/w aqueous dispersion of PPO_60_–PDMAC_120_ nanoparticles.

TEM analysis of PPO_40_–PDMAC_120_ and PPO_60_–PDMAC_120_ confirmed a well-defined spherical morphology with estimated number-average diameters of 104 ± 46 and 121 ± 32 nm, respectively (averaged over 100 nanoparticles, see Figure 6). DLS analysis of PPO–PDMAC nanoparticles at 20 °C indicated unimodal populations in all cases (see Figure S10). Depending on the target diblock copolymer composition, the z-average diameter ranged between 120 and 190 nm. Aqueous electrophoresis studies of the PPO_60_-PDMAC_120_ nanoparticles confirmed that zeta potentials remained approximately zero from pH 4 to pH 9, as expected given the non-ionic nature of the PDMAC steric stabilizer chains (see Figure 7).

Further DLS studies of PPO_60_–PDMAC_120_ nanoparticles conducted at 70 °C indicated thermoresponsive behavior: a population of smaller, more compact nanoparticles with a z-average diameter of 33 nm was observed (Figure S11). Examining the corresponding number-average DLS data, it is apparent that these relatively small nanoparticles vastly outnumber the larger nanoparticles (z-average diameter = 164 nm). This thermoresponsive behavior was confirmed by TEM studies (Figure 6). To evaluate whether this thermoresponsive behavior was reversible, the same PPO_60_–PDMAC_120_ nanoparticles were subjected to three thermal cycles, which involved heating from 20 to 70 °C before returning to 20 °C (see Figure S12). For each cycle, a bimodal population of nanoparticles was observed at 70 °C comprising a major population with a z-average diameter of 164 nm and a minor population with a z-average diameter of 33 nm. After cooling to 20 °C, a unimodal particle size distribution was obtained with a z-average diameter of approximately 163 nm. Given the good reversibility observed over three thermal cycles, this suggests that the amphiphilic diblock copolymer chains form their thermodynamically preferred morphology at each temperature.

On the other hand, freeze-drying an aqueous dispersion of the same PPO_60_–PDMAC_120_ nanoparticles afforded a z-average diameter of 230 nm (PDI = 0.19) after redispersion of the resulting copolymer powder in deionized water at 20 °C (see Figure S13). This is significantly larger than the z-average diameter of 165 nm (PDI = 0.13) obtained for the original PPO_60_–PDMAC_120_ nanoparticles prior to freeze-drying. This suggests that reverse sequence aqueous PISA leads to more well-defined PPO-core nanoparticles compared to that obtained by post-polymerization self-assembly of the copolymer chains.

The core-forming PPO block is known to become more hydrophobic at elevated temperature.^74,75^ These observations are also consistent with our prior studies on closely related PPO-core nanoparticles.^76^ In principle, this reduction in the nanoparticle diameter at elevated temperature suggests further (partial) dehydration of the PPO chains. However, variable temperature ^1^H NMR studies of an aqueous dispersion of PPO_60_–PDMAC_120_ nanoparticles in D_2_O proved to be inconclusive (see Figure S14). More specifically, the integrated proton signal assigned to the methyl protons on the PPO chains remained approximately constant relative to the six pendent dimethyl protons assigned to the PDMAC chains for spectra recorded at 20 and 70 °C. Similar observations were reported by Save et al.^76^

**Figure 8:**
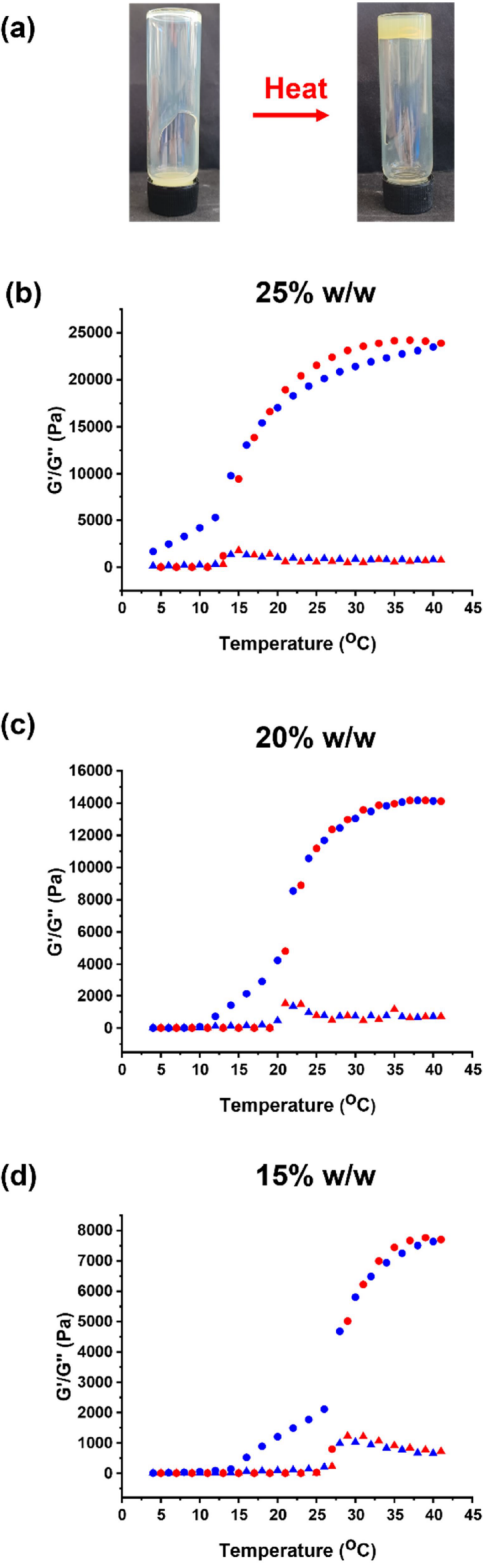
(a) Digital photographs recorded for a 15% w/w aqueous dispersion of PPO_40_–PDMAC_100_ nanoparticles, which form a free-flowing fluid at 20 °C and a free-standing gel at 50 °C. Variable temperature rheological data obtained for aqueous dispersions of PPO_40_−PDMAC_100_ nanoparticles at a copolymer concentration of (b) 25% w/w, (c) 20% w/w, and (d) 15% w/w solids. The temperature dependence of the storage modulus (*G*′, circular data) and loss modulus (*G″*, triangular data) is examined during heating (red data) and cooling (blue data) ramps.

The final copolymer concentration was systematically varied from 5 to 25% w/w solids for the reverse sequence aqueous PISA synthesis of PPO_40_–PDMAC_110_ nanoparticles. The GPC curves for the corresponding diblock copolymers overlap in each case, suggesting almost identical molecular weight distributions (see Figure S15). However, significant differences in the physical appearance of these nanoparticle dispersions were observed (see Figure S16). When a final copolymer concentration of either 5 or 10% w/w solids was achieved, the final copolymer dispersions were free-flowing. In contrast, when dispersions were diluted to 15–25% w/w solids, thermoresponsive gels were obtained. More specifically, rheology studies confirmed that varying the final copolymer concentration enabled the critical gelation temperature to be tuned from 13.0 °C at 25% w/w solids to 27.0 °C at 15% w/w solids (Figure 8). There are many literature studies of the gelation behavior of poly(ethylene oxide)–poly(propylene oxide)–poly(ethylene oxide) (PEO–PPO–PEO) copolymers, otherwise known as *Pluronics*. For example, Booth and co-workers reported that PEO_99_–PPO_69_–PEO_99_ triblock copolymers formed gels at 40 °C for copolymer concentrations above 10% w/w.^77^ Moreover, lower critical gelation temperatures were required at higher copolymer concentrations. Such observations are similar to those made for PPO_40_–PDMAC_100_ diblock copolymers in the present study.

SIPLI analysis of these gels indicated no characteristic Maltese cross motif.^78,79^ This suggests that gelation involves close-packed spherical nanoparticles rather than weakly interacting worms. This was confirmed by diluting such copolymer dispersions to 10% w/w. Under these conditions, no gel was formed on heating because the copolymer concentration is now below the critical concentration required to form close-packed spherical micelle gels.^80^

## Conclusions

We report a new reverse sequence aqueous PISA protocol for the efficient synthesis of thermoresponsive diblock copolymer nanoparticles. For this formulation, DMAC monomer acts as a cosolvent for the weakly hydrophobic PPO precursor. RAFT polymerization of DMAC at 60 °C is initially conducted at 80% w/w solids, followed by dilution to 5–25% w/w. This leads to in situ self-assembly as the PPO chains become insoluble in the reaction mixture, resulting in the formation of aqueous dispersions of diblock copolymer nanoparticles comprising poly(propylene oxide) (PPO) cores and PDMAC coronal chains. Essentially full DMAC conversion is achieved within 16 h, and the diblock copolymer chains exhibit narrow molecular weight distributions (*M*_w_/*M*_n_ ≤ 1.31), suggesting well-controlled RAFT polymerizations. TEM studies confirm a well-defined spherical morphology, while DLS analysis indicates z-average diameters of 120–190 nm. Heating aqueous dispersions of such nanoparticles to 70 °C produces more compact nanoparticles of approximately 30 nm diameter. Tube inversion tests confirm that 15–25% w/w aqueous dispersions of PPO_40_–PDMAC_100_ nanoparticles form thermoresponsive gels at 13 to 27 °C. SIPLI studies suggest that gelation is simply the result of close-packed spheres rather than the formation of anisotropic worm-like nanoparticles. Reversible degelation occurs when the temperature is reduced to 5 °C. Finally, increasing the copolymer concentration lowers the critical degelation temperature, which enables the thermoresponsive behavior to be tuned.

## Supporting Information Available

Summary table of reagent quantities used for polymerizations, additional PPO precursor analysis by NMR and GPC, and additional PPO–PDMAC diblock copolymer analysis by GPC, NMR, and DLS (PDF)
