# Supplementary material for: Synthesis of Poly(propylene oxide)–Poly(N,N′-dimethylacrylamide) Diblock Copolymer Nanoparticles via Reverse Sequence Polymerization-Induced Self-Assembly in Aqueous Solution
Source: Macromolecules. 2023 Dec 17;57(1):317–27. doi: 10.1021/acs.macromol.3c01939 (PMC10782481; doi:10.1021/acs.macromol.3c01939)
Supplement: Supplementary file 1 — ma3c01939_si_001.pdf [file ma3c01939_si_001.pdf]

## Supporting Information for

### *Synthesis of Poly(propylene oxide)-Poly(N,N-dimethylacrylamide) Diblock Copolymer Nanoparticles via Reverse Sequence Polymerization-Induced Self-Assembly in Aqueous Solution*

Matthew A. H. Farmer[a], Osama M. Musa[b], Iris Haug[c], Stefan Naumann[c], and Steven P. Armes[a,*]

*a. Dainton Building, Department of Chemistry, University of Sheffield, Brook Hill, Sheffield, South Yorkshire, S3 7HF, UK.*

*b. Ashland Specialty Ingredients, 1005 US 202/206, Bridgewater, New Jersey, 08807, USA.*

*c. University of Stuttgart, Institute of Polymer Chemistry, 70569 Stuttgart, Germany.*

# Table of Contents

**Table of Reagent Quantities and Intermediate Conversions** ........................................S2

**Supplementary data for the characterization of PPO-TTC percursors** .........................S3

**Supplementary data for the characterization of PPO-PDMAC copolymers**..................S6

## Table of Reagent Quantities and Intermediate Conversions

**Table S1.** Summary of reagent quantities used for DMAC polymerization using either a $PPO_{40}$-TTC or a $PPO_{60}$-TTC precursor. Initially, this polymerization was conducted at high solid concentration, with subsequent dilution via addition of deoxygenated deionized water. Instantaneous DMAC conversions at the time of addition of water were determined via $^1H$ NMR spectroscopy.

| Entry Number | Target copolymer composition | PPO-TTC | DMAC | AIBA | Total Water (Initial Water) | Dilution Time | Intermediate DMAC conversion |
|---|---|---|---|---|---|---|---|
| 1 | $PPO_{40}$-$PDMAC_{160}$ | 0.10 g (0.038 mmol) | 0.60 g (6.03 mmol) | 2.0 mg (7.5 µmol) | 6.30 mL (175 µL) | 85 min | 59 % |
| 2 | $PPO_{40}$-$PDMAC_{140}$ | 0.10 g (0.038 mmol) | 0.52 g (5.28 mmol) | 2.0 mg (7.5 µmol) | 5.63 mL (156 µL) | 71 min | 60 % |
| 3 | $PPO_{40}$-$PDMAC_{120}$ | 0.10 g (0.038 mmol) | 0.45 g (4.52 mmol) | 2.0 mg (7.5 µmol) | 4.95 mL (138 µL) | 40 min | 39 % |
| 4 | $PPO_{40}$-$PDMAC_{100}$ | 0.10 g (0.038 mmol) | 0.37 g (3.77 mmol) | 2.0 mg (7.5 µmol) | 4.28 mL (119 µL) | 41 min | 44 % |
| 5 | $PPO_{40}$-$PDMAC_{80}$ | 0.10 g (0.038 mmol) | 0.30 g (3.01 mmol) | 2.0 mg (7.5 µmol) | 3.61 mL (100 µL) | 49 min | 32 % |
| 6 | $PPO_{40}$-$PDMAC_{60}$ | 0.10 g (0.038 mmol) | 0.22 g (2.26 mmol) | 2.0 mg (7.5 µmol) | 2.94 mL (80 µL) | 40 min | Precipitated |
| 7 | $PPO_{60}$-$PDMAC_{180}$ | 0.10 g (0.038 mmol) | 0.47 g (4.75 mmol) | 2.0 mg (7.5 µmol) | 5.15 mL (143 µL) | 43 min | 51 % |
| 8 | $PPO_{60}$-$PDMAC_{160}$ | 0.10 g (0.026 mmol) | 0.42 g (4.22 mmol) | 1.4 mg (5.3 µmol) | 4.68 mL (130 µL) | 47 min | 46 % |
| 9 | $PPO_{60}$-$PDMAC_{140}$ | 0.10 g (0.026mmol) | 0.37 g (3.69 mmol) | 1.4 mg (5.3 µmol) | 4.21 mL (117 µL) | 50 min | 47 % |
| 10 | $PPO_{60}$-$PDMAC_{120}$ | 0.10 g (0.026 mmol) | 0.31 g (3.16 mmol) | 1.4 mg (5.3 µmol) | 3.74 mL (104 µL) | 80 min | 49 % |
| 11 | $PPO_{60}$-$PDMAC_{100}$ | 0.10 g (0.026 mmol) | 0.26 g (2.64 mmol) | 1.4 mg (5.3 µmol) | 3.27 mL (91 µL) | 110 min | 55 % |
| 12 | $PPO_{60}$-$PDMAC_{80}$ | 0.10 g (0.026 mmol) | 0.21 g (2.11 mmol) | 1.4 mg (5.3 µmol) | 2.79 mL (78 µL) | 200 min | Precipitated |
| 13 | $PPO_{40}$-$PDMAC_{100}$ | 0.10 g (0.038 mmol) | 0.37 g (3.77 mmol) | 2.0 mg (7.5 µmol) | 9.04 mL (119 µL) | 40 min | 34 % |
| 14 | $PPO_{40}$-$PDMAC_{100}$ | 0.10 g (0.038 mmol) | 0.37 g (3.77 mmol) | 2.0 mg (7.5 µmol) | 2.70 mL (119 µL) | 42 min | 48 % |
| 15 | $PPO_{40}$-$PDMAC_{100}$ | 0.10 g (0.038 mmol) | 0.37 g (3.77 mmol) | 2.0 mg (7.5 µmol) | 1.90 mL (119 µL) | 41 min | 50 % |
| 16 | $PPO_{40}$-$PDMAC_{100}$ | 0.10 g (0.038 mmol) | 0.37 g (3.77 mmol) | 2.0 mg (7.5 µmol) | 1.42 mL (119 µL) | 42 min | 37 % |
| 17 | $PPO_{60}$-$PDMAC_{120}$ | 0.10 g (0.026 mmol) | 0.31 g (3.16 mmol) | 1.4 mg (5.3 µmol) | 3.74 mL (46 µL) | 220 min | 32 % |
| 18 | $PPO_{60}$-$PDMAC_{120}$ | 0.10 g (0.026 mmol) | 0.31 g (3.16 mmol) | 1.4 mg (5.3 µmol) | 3.74 mL (178 µL) | 60 min | 52 % |
| 19 | $PPO_{60}$-$PDMAC_{120}$ | 0.10 g (0.026 mmol) | 0.31 g (3.16 mmol) | 1.4 mg (5.3 µmol) | 3.74 mL (277 µL) | 35 min | 68 % |

## Supplementary data for the characterization of PPO-TTC percursors

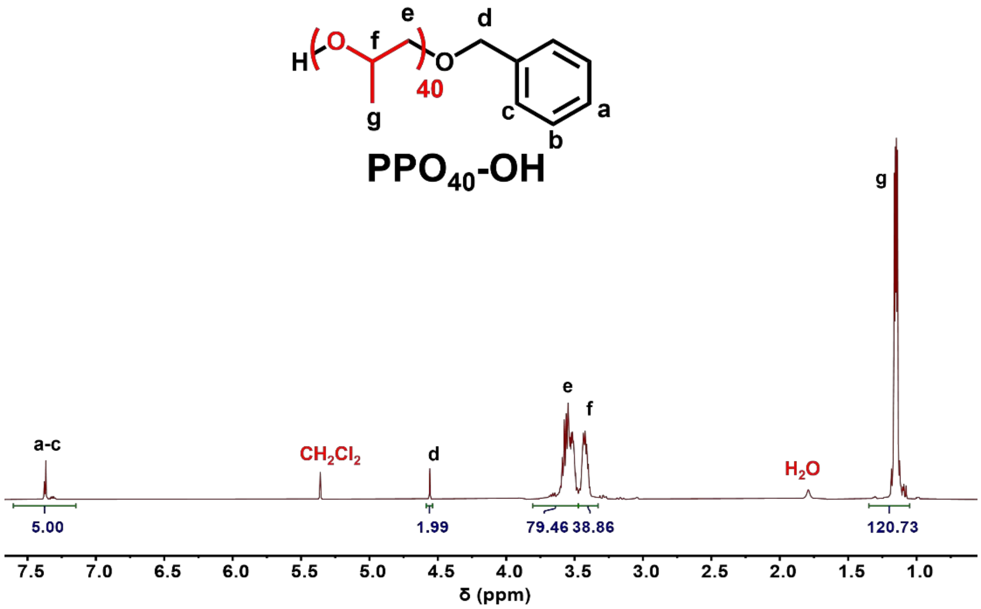


**Figure S1**. Assigned $^{1}$H NMR spectrum ($CD_2Cl_2$) obtained for the $PPO_{40}$-OH precursor. Comparison of the aromatic benzyl proton signals **a-c** at 7.37 ppm with that of the PPO proton signals **g, f** and **e** at 1.16 ppm, 3.43 ppm and 3.55 ppm enabled determination of a mean degree of polymerization of 39.6 ± 0.6 via end-group analysis.

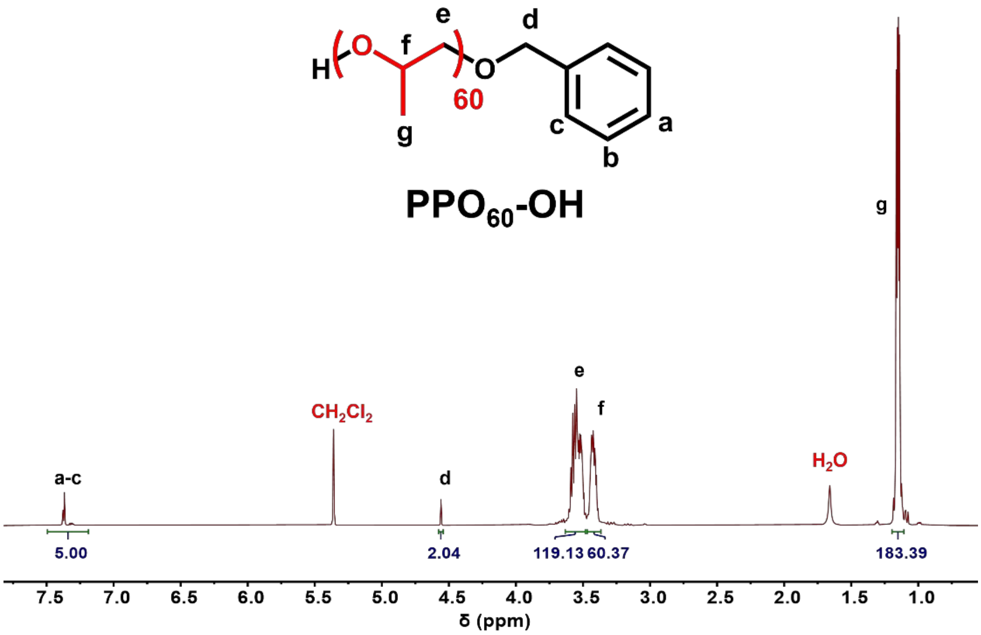


**Figure S2**. Assigned $^{1}$H NMR spectrum ($CD_2Cl_2$) obtained for the $PPO_{60}$-OH precursor. Comparison of the aromatic benzyl proton signals **a-c** at 7.37 ppm with that of the PPO proton signals **g, f** and **e** at 1.16 ppm, 3.43 ppm and 3.55 ppm enabled determination of a mean degree of polymerization of 60.4 ± 0.6 via end-group analysis.

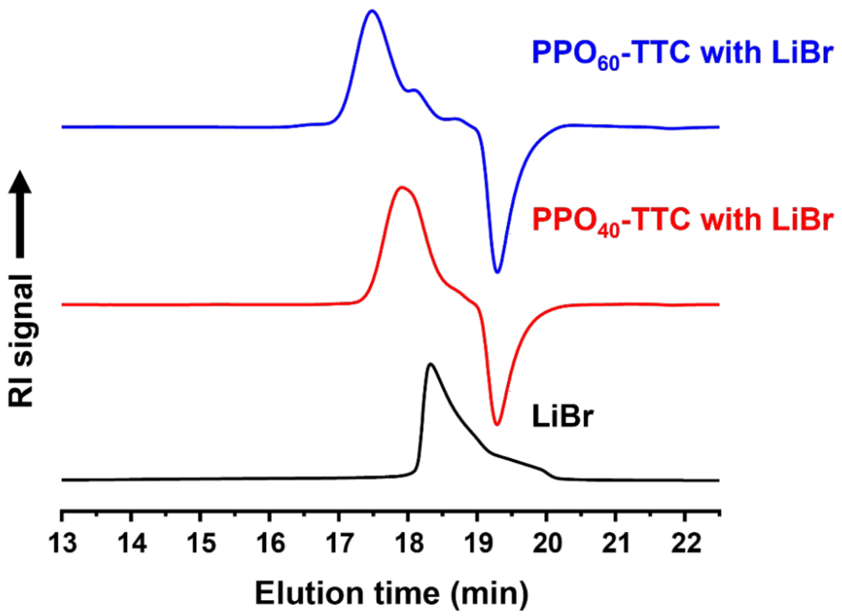


**Figure S3.** DMF GPC curves (refractive index detector) recorded for $PPO_{60}$-TTC (blue curve) and $PPO_{40}$-TTC (red curve) precursors in the presence of LiBr. DMF GPC curve recorded in the absence of any copolymer with LiBr additive in the eluent (black curve).

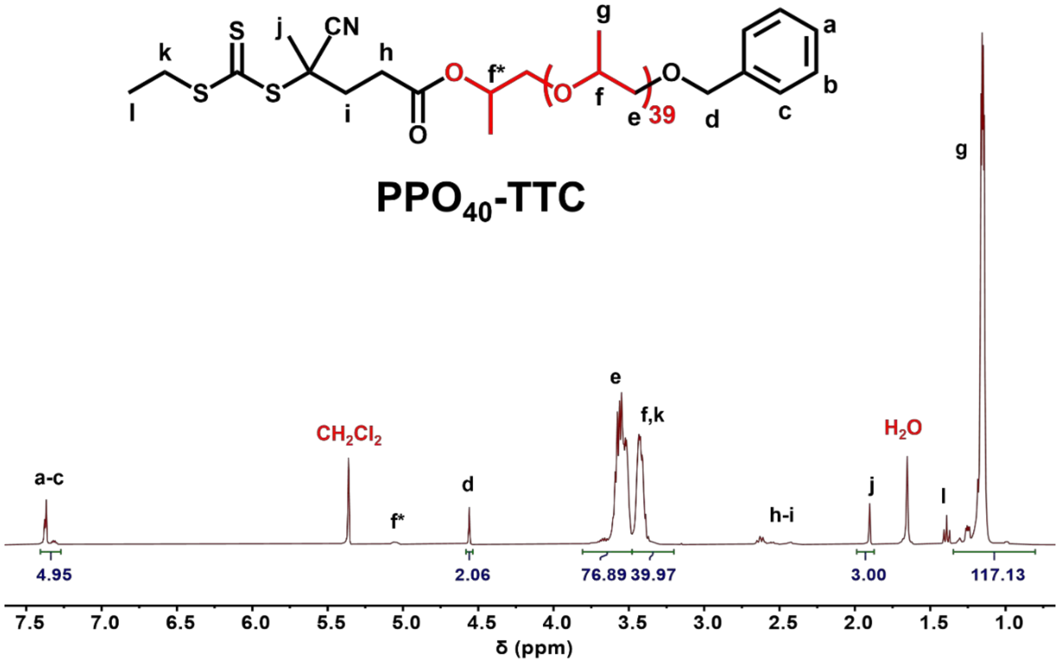


**Figure S4**. Assigned $^{1}H$ NMR spectrum ($CD_2Cl_2$) of the functionalized $PPO_{40}$-TTC precursor. Comparison of the integrated proton signal **j** at 1.91 ppm assigned to the methyl group of the RAFT agent with that of the unique PPO proton signals **e** and **g** at 1.16 ppm and 3.55 enabled determination of a mean degree of esterification of 97 ± 1 %.

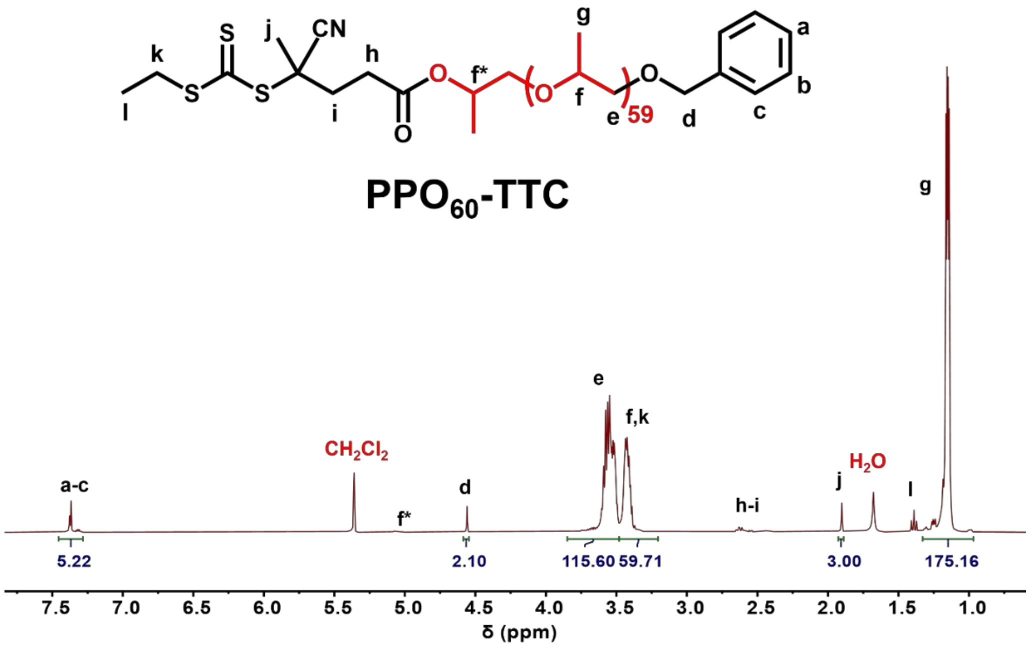


**Figure S5**. Assigned $^{1}$H NMR spectrum ($CD_2Cl_2$) of the functionalized $PPO_{60}$-TTC precursor. Comparison of the integrated proton signal **j** at 1.91 ppm assigned to the methyl group of the RAFT agent with that of the unique PPO proton signals **e** and **g** at 1.16 ppm and 3.55 enabled determination of a mean degree of esterification of 97 ± 1 %.

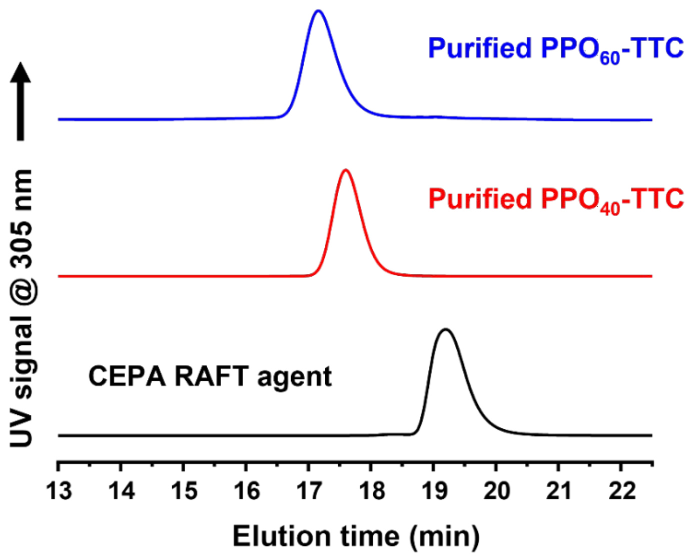


**Figure S6.** DMF GPC curves (UV detector set at λ = 305 nm) recorded for the CEPA RAFT agent (black curve) and the $PPO_{60}$-TTC (blue curve) and $PPO_{40}$-TTC (red curve) precursors. GPC analysis of CEPA required addition of glacial acetic acid (but not LiBr) to the DMF eluent: this prevented ionization of the carboxylic acid group of this RAFT agent.

## Supplementary data for the characterization of PPO-PDMAC copolymers

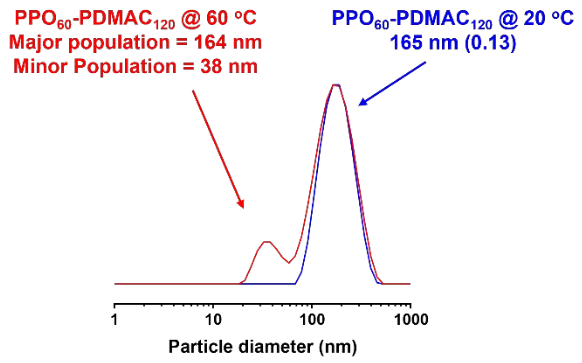


**Figure S7.** Intensity-average particle size distributions obtained by DLS analysis of a 1% w/w aqueous dispersion of $PPO_{60}$-$PDMAC_{120}$ nanoparticles at 20 °C (blue data) and 60 °C (red data).

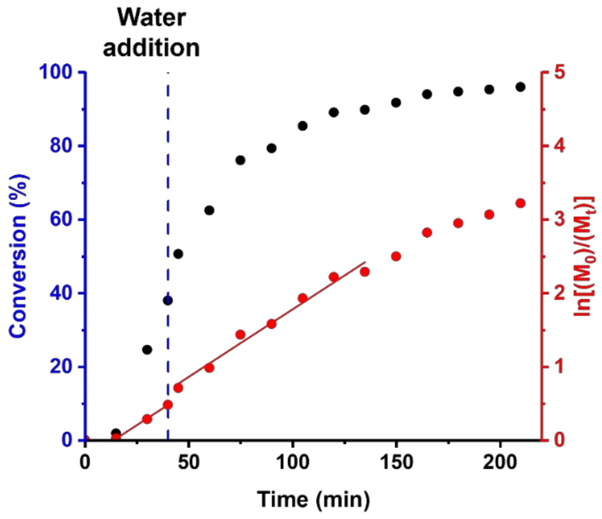


**Figure S8.** Conversion vs. time curve (black points) and the corresponding semi-logarithmic plot (red points) obtained by $^{1}$H NMR spectroscopy for the *reverse sequence* aqueous PISA synthesis of $PPO_{40}$-$PDMAC_{120}$ nanoparticles at 60 °C. Initially, the RAFT polymerization of DMAC was conducted at 80% w/w solids with subsequent dilution to 10% w/w solids using deoxygenated deionized water after 40 min (which corresponds to 38% DMAC conversion). A final DMAC conversion of 96% was achieved within 210 min at 60 °C. Conditions: [$PPO_{40}$-TTC]/[AIBA] molar ratio = 5.0. Before and after the dilution point the gradient of the semi-log plot is equal to 0.0183.

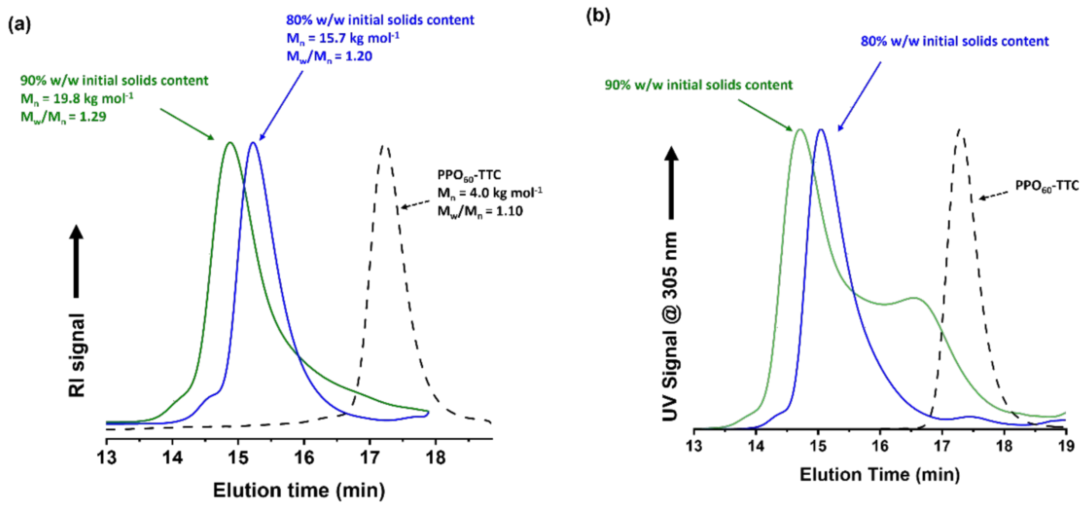


**Figure S9.** DMF GPC curves recorded using (a) a refractive index detector and (b) a UV detector set at λ = 305 nm for a $PPO_{60}$-$PDMAC_{120}$ diblock copolymer prepared by *reverse sequence* aqueous PISA initially at 80 or 90% w/w solids content at 60 °C and a $PPO_{60}$-TTC precursor. Conditions: [$PPO_n$-TTC]/[AIBA] molar ratio = 5.0. [N.B. Diblock copolymer GPC curves are only shown up to an elution time of 17.9 min to omit the signal attributed to LiBr (see **Figure S3**). GPC analysis of the PPO precursor was performed in the absence of any LiBr salt].

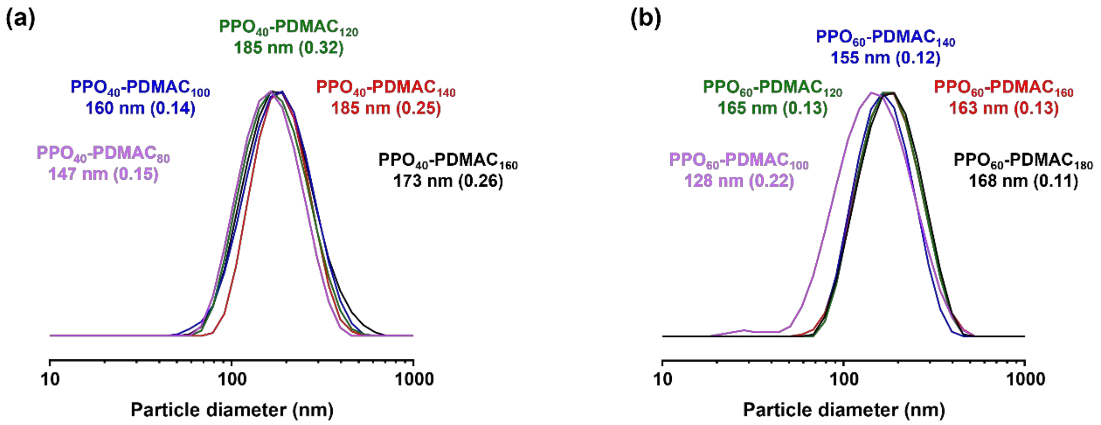


**Figure S10.** DLS particle size distribution recorded for 1% w/w aqueous dispersions of (a) $PPO_{40}$-$PDMAC_m$ nanoparticles and (b) $PPO_{60}$-$PDMAC_m$ nanoparticles prepared via *reverse sequence* PISA.

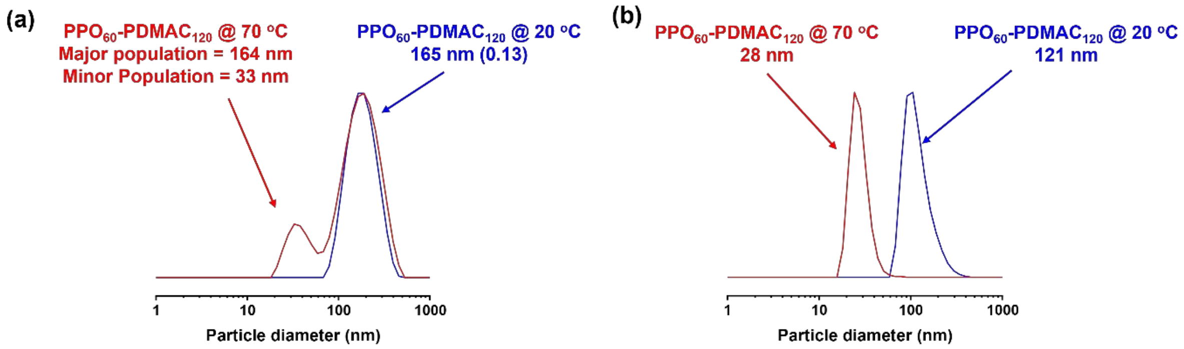


**Figure S11.** (a) Intensity-average and (b) number-average particle size distributions obtained by DLS analysis of a 1% w/w aqueous dispersion of $PPO_{60}$-$PDMAC_{120}$ nanoparticles at 20 °C (blue data) and 70 °C (red data).

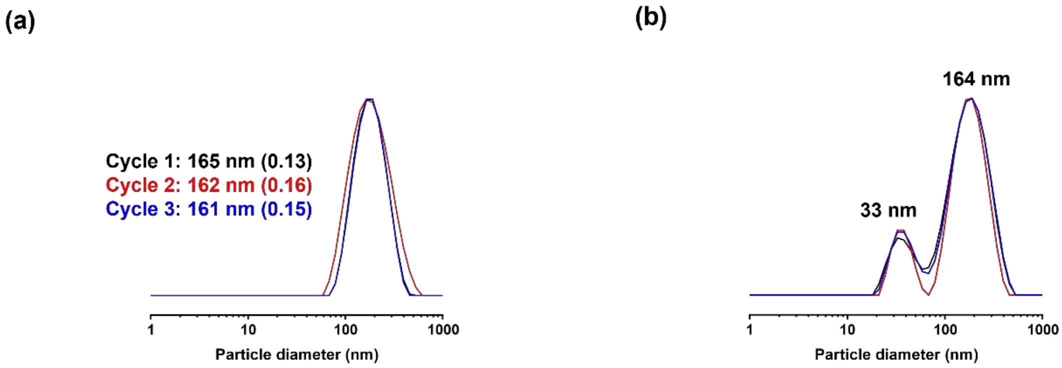


**Figure S12.** DLS analysis of $PPO_{60}$-$PDMAC_{120}$ nanoparticles at (a) 20 °C and (b) 70 °C where the nanoparticles were subjected to three thermal cycles, heating from 20 °C to 70 °C before returning to 20 °C.

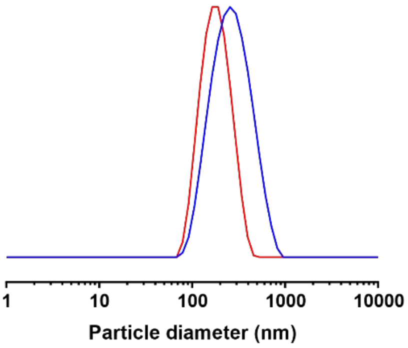


**Figure S13.** DLS analysis of $PPO_{60}$-$PDMAC_{120}$ nanoparticles prior to (red data) and after freeze-drying and redispersal in deionized water at 1% w/w solids (blue data).

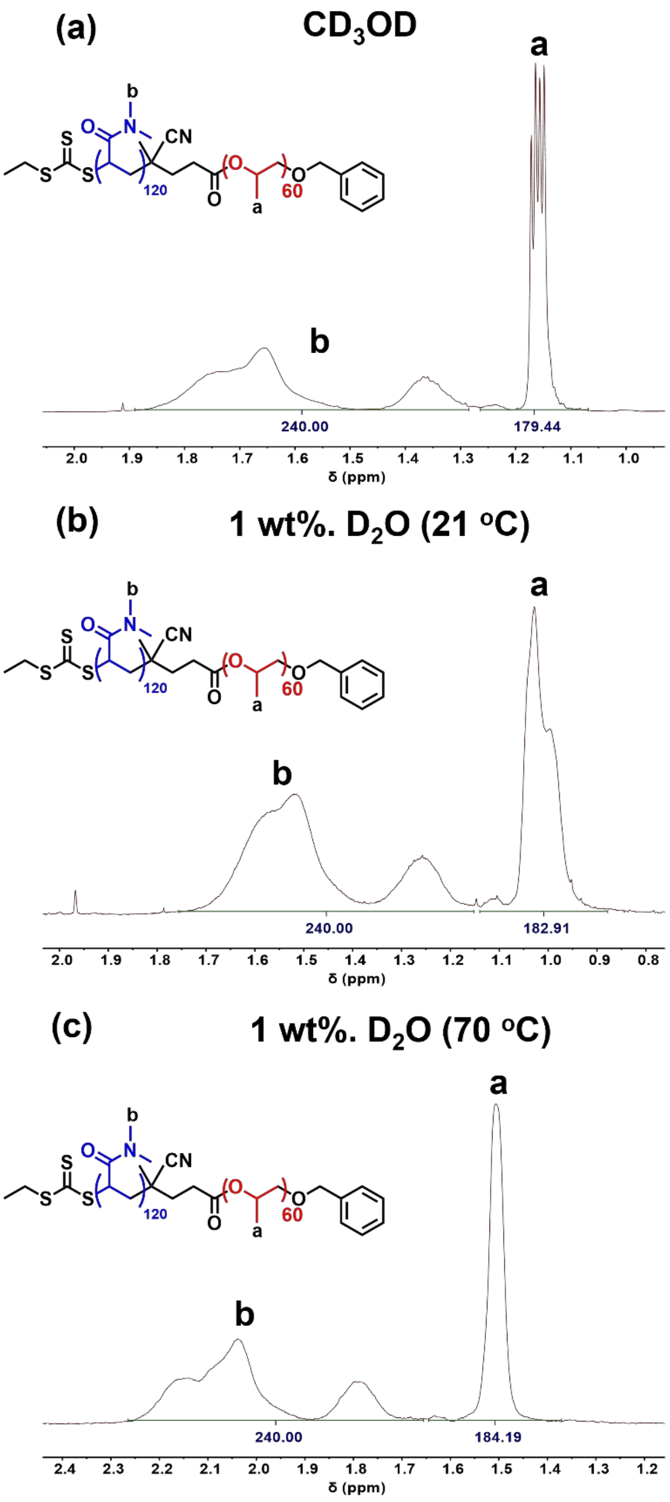


**Figure S14.** $^{1}$H NMR spectra recorded for $PPO_{60}$-$PDMAC_{120}$ in (a) $CD_3OD$, (b) in $D_2O$ at 21 $^{o}$C, and (c) in $D_2O$ at 70 $^{o}$C. The integrated intensity of the signals assigned to the pendant methyl group of the core-forming PPO block at 1.00-1.50 ppm are compared to the methyl groups of the corona-forming PDMAC signals at 1.30-2.30 ppm.

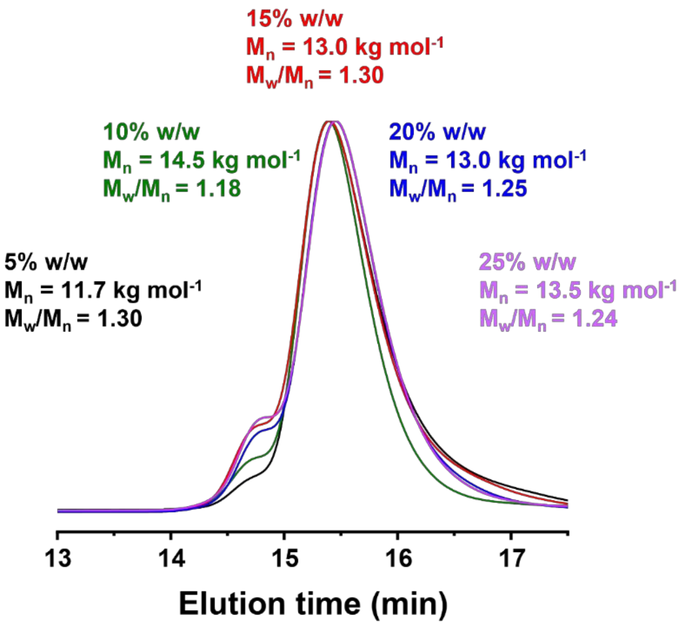


**Figure S15.** DMF GPC curves (refractive index detector) obtained for five $PPO_{40}$-$PDMAC_{100}$ diblock copolymers synthesized via *reverse sequence* PISA (initially at 80% w/w solids followed by dilution to 5-25% w/w at intermediate DMAC conversion).

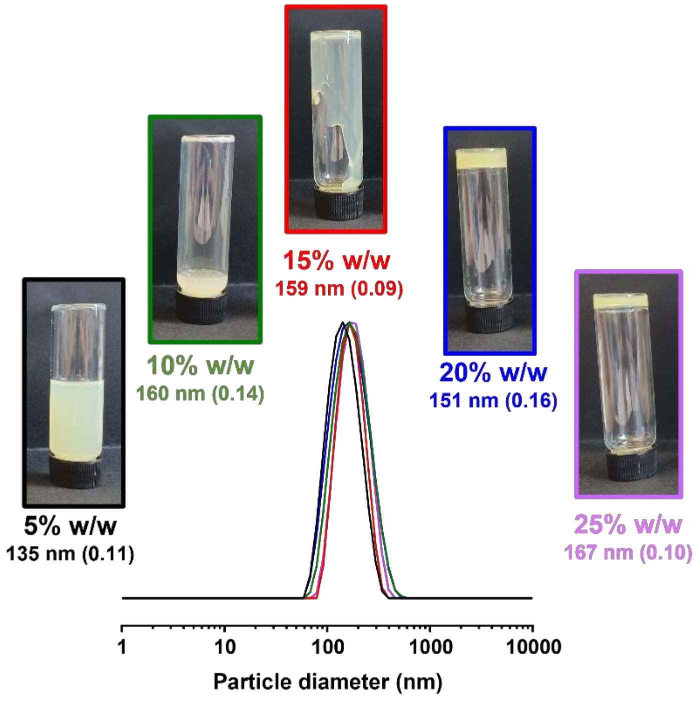


**Figure S16.** DLS particle size distributions recorded for five 1.0% w/w aqueous dispersions of $PPO_{40}$-$PDMAC_{100}$ diblock copolymer nanoparticles prepared via *reverse sequence* PISA (initially at 80% w/w solids followed by dilution to 5-25% w/w at intermediate DMAC conversion).
